# Risks to Health Among American Indian/Alaska Native High School Students in the United States

**Published:** 2011-06-15

**Authors:** Sherry Everett Jones, Khadija Anderson, Richard Lowry, Holly Conner

**Affiliations:** Division of Adolescent and School Health, Centers for Disease Control and Prevention; Care Resource, Inc, Miami, Florida; Centers for Disease Control and Prevention, Atlanta, Georgia; Centers for Disease Control and Prevention, Atlanta, Georgia

## Abstract

**Introduction:**

According to the World Health Organization, the 10 leading risk factor causes of death in high-income countries are tobacco use, high blood pressure, overweight and obesity, physical inactivity, high blood glucose, high cholesterol, low fruit and vegetable intake, urban air pollution, alcohol use, and occupational risks. We examined the prevalence of some of the leading risks to health among nationally representative samples of American Indian/Alaska Native (AI/AN) high school students and compared rates across racial/ethnic groups.

**Methods:**

We combined data from the 2001, 2003, 2005, 2007, and 2009 national Youth Risk Behavior Survey. The survey is a biennial, self-administered, school-based survey of 9th- through 12th-grade students in the United States. Overall response rates for the surveys ranged from 63% to 72%. Of 73,183 participants, 952 were AI/AN students.

**Results:**

For 7 of the 16 variables examined in this study, the prevalence among AI/AN high school students was higher than the prevalence among white high school students. For 1 variable (ate fruit and vegetables <5 times per day), the prevalence among AI/AN students was significantly lower than that among white students. The prevalence for the remaining 8 variables was similar among AI/AN students and white students. These findings also show differences in the prevalence of some behaviors among AI/AN, black, and Hispanic students.

**Conclusion:**

These findings show the prevalence of some health risk behaviors was significantly higher among AI/AN high school students than among high school students in other racial/ethnic groups.

## Introduction

American Indians and Alaska Natives (AI/ANs) are people who have origins in any of the original peoples of North America and who maintain cultural identification through tribal affiliation or community recognition (1). In the 2000 Census, AI/ANs comprised approximately 1.5% of the US population (2). Approximately 2.5 million people identified only American Indian or Alaska Native as their race, and an additional 1.6 million people reported American Indian and at least 1 other race (2). As of 2000, 33% of the AI/AN population was younger than 18 years old, compared with only 26% of the total US population (3). In the 2005-2006 school year, 644,000 AI/AN youth attended public schools (4).

AI/ANs have higher rates of illness and death than do members of other US racial/ethnic groups (5-8). For example, the prevalence of heart disease and diabetes is higher among AI/AN adults than among adults in any other racial/ethnic group (5,9), and rates of physical inactivity (5,9), obesity (5,9,10), and cigarette smoking (5,9,10) are higher than those among white adults.

National (11-16), regional (17,18), and local (19) data show that AI/AN youth are at greater risk for many health problems than their non-AI/AN peers. Previous studies of disparities in health risk between AI/AN youth and youth of other racial/ethnic groups have been limited by a lack of adequate national data (20,21) and have either examined a limited number of risk behaviors or been based on data from limited groups of AI/AN youth.

According to the World Health Organization (WHO), the 10 leading risk factor causes of death in high-income countries are are tobacco use, high blood pressure, overweight and obesity, physical inactivity, high blood glucose, high cholesterol, low fruit and vegetable intake, urban air pollution, alcohol use, and occupational risks (22). We examined the prevalence of some of the leading risks to health among a nationally representative sample of US high school students and identified differences in prevalence among AI/AN students and students of other racial/ethnic groups. We also examined differences in prevalence of those health risks by sex among AI/AN students.

## Methods

### Sample and survey administration

The Centers for Disease Control and Prevention (CDC) developed the Youth Risk Behavior Surveillance System to monitor priority health risk behaviors among youth over time. The national school-based Youth Risk Behavior Survey (YRBS) is a cross-sectional study that has been conducted biennially since 1991. In each survey year, a similar independent 3-stage cluster sample design is used to obtain a nationally representative sample of public and private school students in grades 9 through 12 in the 50 states and the District of Columbia. The YRBS sampling frame, however, does not include schools funded by the Bureau of Indian Education (which serves fewer than 10% of all AI/AN students enrolled in elementary and secondary schools). To generate a sufficient number of AI/AN students, we combined YRBS data from the 2001, 2003, 2005, 2007, and 2009 survey years.

Student participation in the YRBS is anonymous and voluntary, and the YRBS is conducted in accordance with local parental permission procedures. CDC's institutional review board approved the protocol for the national YRBS. YRBS participants complete a self-administered questionnaire during a regular class period and record their responses in a computer-scannable questionnaire booklet. We did not impute values for missing data. For the survey years, school response rates ranged from 75% to 81%, student response rates ranged from 83% to 88%, overall response rates ranged from 63% to 72%, and sample sizes ranged from 13,601 to 16,410. We applied a weighting factor to each record to adjust for school and student nonresponse and oversampling of black and Hispanic students. Details of the YRBS sampling strategies and the psychometric properties of the YRBS questionnaire have been reported elsewhere (23,24).

### Measures

The YRBS measures 6 categories of health risk behaviors: 1) behaviors that contribute to unintentional injuries and violence; 2) tobacco use; 3) alcohol and other drug use; 4) sexual behaviors that contribute to unintended pregnancy and sexually transmitted disease, including human immunodeficiency virus infection; 5) unhealthy dietary behaviors; and 6) physical inactivity. The YRBS also monitors rates of obesity and overweight on the basis of body mass index (BMI) estimates derived from students’ self-reported height and weight. In this study, we focused on the prevalence of variables measured in the YRBS that are consistent with behaviors or conditions WHO cites as the leading health risks (22): cigarette and alcohol use, dietary behaviors, overweight and obesity, and physical inactivity.

The total unweighted sample size for the combined 2001, 2003, 2005, 2007, and 2009 national YRBS surveys was 73,183. For this analysis, the sample included 952 AI/AN students (0.9% [weighted percent]), 15,314 black students (14.2% [weighted percent]), 19,111 Hispanic students (15.9% [weighted percent]), and 31,707 white students (61.8% [weighted percent]). Data from 1,098 students whose race/ethnicity was not indicated and from 5,001 students in the "other" racial/ethnic category were excluded from this analysis.

### Analysis

To account for the complex sample design of the survey, we conducted all analyses on weighted data by using SUDAAN version 9.0.1 (Research Triangle Institute, Research Triangle Park, North Carolina). Because the sex and age distributions in the 4 racial/ethnic groups were similar, we used *t *tests to identify significant differences between prevalence estimates for AI/AN students and those for students of other racial/ethnic groups; *t *tests were also used to identify significant differences in prevalence estimates between AI/AN girls and AI/AN boys. We considered differences significant at *P* ≤ .05.

## Results

### Cigarette and alcohol use

The prevalence of ever having smoked cigarettes and current cigarette use was higher among AI/AN students than students in any other racial/ethnic group (Table 1). The prevalence of current frequent cigarette use was significantly higher among AI/AN students than among black and Hispanic students. The prevalence of each of the 3 alcohol use behaviors was higher among AI/AN students than among black students. The prevalence of current alcohol use and binge drinking was higher among AI/AN boys than AI/AN girls.

### Dietary behaviors, obesity, and overweight

Although the prevalence of eating fruits and vegetables fewer than 5 times per day was higher among white students than among AI/AN students, the prevalence of overweight and obesity was higher among AI/AN students than among white students (Table 2). The prevalence of drinking less than 3 glasses per day of milk was higher among black and Hispanic students than among AI/AN students. The prevalence of drinking less than 3 glasses per day of milk was higher among AI/AN girls than among AI/AN boys, whereas the prevalence of obesity was significantly higher among AI/AN boys than among AI/AN girls.

### Physical activity

The prevalence of insufficient vigorous physical activity, watching television 3 or more hours per day, and not playing on at least 1 sports team (run by their school or community groups) was higher among AI/AN students than among white students (Table 3). The prevalence of insufficient vigorous physical activity was higher among black students than among AI/AN students. The prevalence of insufficient moderate physical activity and watching television 3 or more hours per day was higher among both black and Hispanic students than among AI/AN students. The prevalence of not attending a physical education class was higher among AI/AN students than among Hispanic students. The prevalence of insufficient vigorous physical activity and not playing on at least 1 sports team was higher among AI/AN girls than among AI/AN boys. The prevalence of using computers 3 or more hours per day on an average school day was higher among AI/AN boys than among AI/AN girls.

## Discussion

The findings of this analysis of YRBS data are generally consistent with national (11-16), regional (17,18), and local (19) data showing that AI/AN youth are at greater risk for many health problems than their non-AI/AN peers. This study fills a gap in the public health literature by providing nationally representative data of AI/AN, white, black, and Hispanic US high school students for various behaviors associated with the leading risks to health. For 7 of the 16 variables examined in this study, the prevalence among AI/AN high school students was higher than the prevalence among white high school students. The prevalence of all smoking and alcohol use variables was higher among AI/AN students than among black students, and the prevalence of all smoking variables and not attending physical education classes was higher among AI/AN students than among Hispanic students.

The causes of disparities in health outcomes are complex. Poor health outcomes are associated not only with engaging in health risk behaviors but also with poverty, unemployment, low education levels, and limited access to health care (26), all of which are common to many AI/AN communities (6,27,28). For example, an estimated 1 in 4 AI/AN adults lives in poverty compared with 1 in 11 white adults (5), and an estimated 16% of AI/AN children have no health insurance compared with 6% of white children (6).

The findings from this study suggest that public health action will be needed to reduce the prevalence of health risk behaviors among AI/AN students. There are more than 560 federally recognized AI/AN tribes in the United States (4). The populations of these tribes range from fewer than 100 people to more than 750,000 people (20), and each tribe has its own distinct traditions and cultural heritage (17,27,29). Understanding the geographic, legal, and cultural context in which AI/AN people live is essential to addressing the elevated risk for many health problems found among AI/ANs: a one-size-fits-all approach would not be appropriate. Any school or community-based intervention should be conducted in partnership with AI/AN people to ensure that it is culturally relevant and includes development of the community capacity necessary to ensure the sustainability of the intervention (17,28).

In addition to the importance of culturally relevant interventions, for some behaviors, differences in the prevalence among boys and girls should be considered. For example, AI/AN girls drank less milk than AI/AN boys, and understanding this difference is important for ensuring adequate calcium intake among girls. Likewise, more AI/AN girls than boys had insufficient vigorous physical activity and did not play on a sports team. In contrast, the prevalence of binge drinking, computer use, and obesity was higher among AI/AN boys than girls.

The public health goals for the nation set in *Healthy People 2010* address, in part, health risk behaviors among youth (26). The findings of this analysis of YRBS data show that AI/AN high school students (and students in other racial/ethnic groups) have not yet met all of the target rates set in the *Healthy People 2010* goals (Table 4). For example, the 2010 goal for current cigarette use is 16%, but 33.7% of AI/AN students were current cigarette users. The prevalence among black students was 12.6%, lower than the *Healthy People 2010* goal. The goal for binge drinking is 2.0%, but the prevalence of binge drinking among AI/AN students was 30.9% (nearly equivalent to the prevalence among white and Hispanic students). Similarly, the targets for vigorous physical activity (85% goal) and moderate physical activity (35% goal) have not been met, and rates among AI/AN students are 62.4% and 28.1%, respectively (ie, the inverses of insufficient vigorous and moderate physical activity reported in this study). A similar pattern is seen with other *Healthy People 2010* goals for comparable risk behaviors measured in this study.

The findings of this study should be considered in the context of some limitations. First, because of the small number of students who identified their race as AI/AN, multiple years of YRBS data were combined to produce meaningful estimates of the prevalence of health risk behaviors. As a result, these prevalence estimates do not reflect any changes in the prevalence of these factors that may have occurred during the study period. In addition, because of the small number of AI/AN students in this study (even after combining 5 survey years of data), the 95% confidence intervals around the prevalence estimates for AI/AN students are wide. Second, because the YRBS combines American Indian and Alaska Native into a single race category, it was not possible to produce separate prevalence estimates for each group, and the results of at least 1 study showed notable differences in the prevalence of some risk behaviors among Alaska Native youth compared with American Indian youth not living in Alaska (17). Third, these data apply only to adolescents who attend high school and do not capture students who have dropped out of school. Nationwide, in 2007, of people aged 16 to 17 years, approximately 4% were not enrolled in a high school program and had not completed high school (30); the dropout rate among AI/AN youth is higher than among white and black youth, though lower than among Hispanic youth (4). Fourth, because the sampling frame did not include schools funded by the Bureau of Indian Education, the findings from this study are not generalizable to students served by those schools.

Assessment of the effectiveness of efforts to reduce or eliminate health disparities in the United States, a focus of *Healthy People 2010* (26), requires ongoing analyses of high-quality surveillance data from minority populations. This analyses of YRBS data found that the prevalence of some health risk behaviors was significantly higher among AI/AN high school students than among high school students in other racial/ethnic groups, indicating that they are at higher risk for illness and premature death than students in other racial/ethnic groups. Public health efforts targeted to this population are essential to addressing these disparities.

## Figures and Tables

**Table 1:** Percentage of High School Students Who Smoked Cigarettes and Drank Alcohol, by Race/Ethnicity — United States, Youth Risk Behavior Survey, 2001-2009

**Behavior**	AI/AN,^a^^,^ b % (95% CI)	White,^a^ % (95% CI)	Black,^a^ % (95% CI)	Hispanic, % (95% CI)
**Ever smoked cigarettes^c^**				
Total	71.2 (66.9-75.1)^W,B,H^	54.6 (52.8-56.4)	52.6 (50.7-54.4)	57.5 (55.5-59.5)
Girls	70.5 (64.5-75.9)^W,B,H^	54.1 (52.3-55.9)	51.4 (49.1-53.7)	54.9 (52.8-56.9)
Boys	71.6 (65.7-76.9)^W,B,H^	55.1 (52.8-57.4)	53.7 (51.6-55.8)	60.2 (57.7-62.6)
**Current cigarette use^d^**				
Total	33.7 (29.3-38.3)^W,B,H^	25.7 (24.5-26.9)	12.6 (11.7-13.6)	19.8 (18.3-21.3)
Girls	33.5 (28.4-38.9)^W,B,H^	26.1 (24.7-27.5)	10.4 (9.4-11.5)	18.3 (16.9-19.7)
Boys	33.5 (27.4-40.2)^W,B,H^	25.3 (23.9-26.7)	14.8 (13.5-16.3)	21.3 (19.3-23.4)
**Current frequent cigarette use^e^**				
Total	14.0 (10.5-18.5)^B,H^	12.0 (11.3-12.8)	3.9 (3.3-4.5)	5.3 (4.7-6.0)
Girls	12.5 (8.3-18.6)^B,H^	12.2 (11.2-13.2)	2.4 (1.9-2.9)	4.1 (3.5-4.8)
Boys	15.4 (11.0-21.1)^B,H^	11.9 (11.1-12.8)	5.5 (4.6-6.5)	6.5 (5.6-7.5)
**Ever drank alcohol^f^**				
Total	78.8 (73.9-83.0)^B^	76.1 (74.6-77.5)	69.2 (67.6-70.7)	78.5 (77.2-79.8)
Girls	78.8 (73.2-83.5)^B^	76.8 (75.2-78.3)	71.0 (69.1-72.9)	79.6 (78.1-81.0)
Boys	78.6 (72.0-84.0)^B^	75.4 (73.8-77.0)	67.2 (65.4-69.0)	77.6 (76.0-79.1)
**Current alcohol use^g^**				
Total	48.2 (42.8- 53.7)^B^	47.2 (45.9-48.5)	33.9 (32.5-35.3)	46.1 (44.7-47.4)
Girls	42.6 (36.8-48.7)^B^	47.2 (45.7-48.6)	34.2 (32.5-36.0)	46.4 (44.8-48.0)
Boys	53.1 (44.8-61.3)^G,B^	47.2 (45.6-48.8)	33.4 (31.6-35.2)	45.7 (43.9-47.5)
**Binge drinking^h^**				
Total	30.9 (26.0-36.3)^B^	30.7 (29.7-31.7)	12.9 (12.0-13.8)	26.7 (25.5-28.1)
Girls	26.3 (20.7-32.8)^B^	29.1 (28.1-30.2)	10.8 (9.8-11.8)	25.6 (24.3-27.0)
Boys	34.8 (28.0-42.2)^G,B^	32.1 (30.7-33.6)	15.0 (13.7-16.4)	27.9 (26.1-29.7)

Abbreviations: AI/AN, American Indian/Alaska Native; CI, confidence interval.

a Non-Hispanic.

b The letter W (white), B (black), or H (Hispanic) indicates a significant difference in prevalence estimates between AI/AN students and students of that race/ethnicity. The letter G indicates a significant difference in prevalence estimates between AI/AN girls and boys (*P* ≤ .05, *t* test).

c Ever tried cigarette smoking, even 1 or 2 puffs.

d Smoked cigarettes on at least 1 day during the 30 days before the survey.

e Smoked cigarettes on 20 or more days during the 30 days before the survey.

f Had at least 1 drink of alcohol on at least 1 day during their life.

g Had at least 1 drink of alcohol on at least 1 day during the 30 days before the survey.

h Had 5 or more drinks of alcohol in a row, within a couple of hours, on at least 1 day during the 30 days before the survey.

**Table 2:** Percentage of High School Students Who Engaged in Certain Dietary Behaviors, Were Overweight, or Were Obese, by Race/Ethnicity — United States, Youth Risk Behavior Survey, 2001-2009

**Behavior or Characteristic**	AI/AN,^a^^,^ b % (95% CI)	White,^a^ % (95% CI)	Black,^a^ % (95% CI)	Hispanic, % (95% CI)
**Ate fruits and vegetables <5 times/d^c^**				
Total	73.5 (68.3-78.1)^W^	80.2 (79.4-81.0)	75.6 (74.5-76.7)	76.7 (75.6-77.8)
Girls	76.7 (68.7-83.2)	81.4 (80.3-82.4)	77.9 (76.6-79.2)	79.0 (77.6-80.3)
Boys	70.3 (65.0-75.2)^W^	79.2 (78.3-80.1)	73.3 (71.7-74.8)	74.5 (72.8-76.0)
**Drank <3 glasses/d of milk^d^**				
Total	83.3 (79.4-86.6)^B,H^	82.0 (80.7-83.2)	89.9 (89.1-90.6)	87.1 (86.2-88.0)
Girls	90.0 (85.5-93.2)^B^	88.3 (87.0-89.4)	93.8 (93.0-94.5)	91.5 (90.7-92.3)
Boys	77.2 (71.5-82.0)^G,B,H^	76.0 (74.4-77.5)	85.8 (84.5-87.0)	82.8 (81.4-84.1)
**Overweight^e^**				
Total	20.0 (17.0-23.4)^W^	13.6 (13.0-14.2)	19.2 (18.3-20.2)	17.8 (17.0-18.7)
Girls	21.3 (15.9-28.0)^W^	12.4 (11.7-13.2)	21.3 (19.8-22.8)	17.4 (16.3-18.6)
Boys	18.9 (15.2-23.1)^W^	14.7 (14.0-15.5)	17.2 (15.9-18.6)	18.2 (17.2-19.3)
**Obese^f^**				
Total	15.5 (12.7-18.9)^W^	10.4 (9.8-11.0)	16.3 (15.5-17.2)	16.0 (15.0-17.0)
Girls	10.1 (7.0-14.3)^B^	6.6 (6.0-7.3)	15.0 (13.8-16.3)	11.4 (10.4-12.4)
Boys	20.4 (16.5-24.9)^G,W^	14.0 (13.1-14.9)	17.6 (16.5-18.9)	20.5 (19.1-21.9)

Abbreviations: AI/AN, American Indian/Alaska Native; CI, confidence interval.

a Non-Hispanic.

b The letter W (white), B (black), or H (Hispanic) indicates a significant difference in prevalence estimates between AI/AN students and students of that race/ethnicity. The letter G indicates a significant difference in prevalence estimates between AI/AN girls and boys (*P* ≤ .05, *t* test).

c 100% fruit juice, fruit, green salad, potatoes (excluding French fries, fried potatoes, or potato chips), carrots, or other vegetables during the 7 days before the survey.

d During the 7 days before the survey (includes milk drunk in a glass or cup, from a carton, or with cereal).

e Students who were ≥85th percentile but <95th percentile for body mass index, by age and sex, based on reference data from Centers for Disease Control and Prevention growth charts (25).

f Students who were ≥95th percentile for body mass index, by age and sex, based on reference data from Centers for Disease Control and Prevention growth charts (25).

**Table 3:** Percentage of High School Students Who Engaged in Physical Activity-Related Behaviors, by Race/Ethnicity — United States, Youth Risk Behavior Survey, 2001-2009

**Activity**	AI/AN,^a^^,^ b % (95% CI)	White,^a^ % (95% CI)	Black,^a^ % (95% CI)	Hispanic, % (95% CI)
**Insufficient vigorous physical activity^c^**				
Total	37.6 (34.0-41.4)^W,B^	33.1 (31.9-34.4)	41.9 (40.5-43.3)	37.2 (35.8-38.5)
Girls	44.8 (39.3-50.4)^B^	40.2 (38.6-41.8)	52.7 (50.8-54.5)	45.9 (44.0-47.8)
Boys	31.1 (25.9-36.8)^G^	26.3 (25.0-27.8)	30.7 (29.2-32.4)	28.4 (26.8-30.1)
**Insufficient moderate physical activity^d^**				
Total	71.9 (68.2-75.2)^B,H^	71.9 (71.0-72.7)	78.4 (77.3-79.4)	76.5 (75.4-77.6)
Girls	72.5 (66.1-78.1)^B,H^	74.8 (73.7-75.8)	82.3 (80.8-83.6)	79.0 (77.7-80.3)
Boys	71.0 (65.7-75.8)	69.1 (68.2-70.0)	74.4 (72.9-75.7)	74.1 (72.6-75.6)
**Watched television >3 hours/d^e^**				
Total	38.6 (33.2-44.3)^W,B,H^	28.3 (27.3-29.3)	63.3 (61.9-64.6)	44.5 (42.8-46.2)
Girls	35.2 (27.7-43.6)^W,B,H^	25.6 (24.4-26.8)	63.9 (62.1-65.7)	44.1 (42.5-45.7)
Boys	41.9 (36.3-47.7)^W,B^	30.8 (29.5-32.2)	62.6 (60.8-64.3)	45.0 (42.6-47.3)
**Used computers >3 hours/d^e^^,^ f**				
Total	25.5 (21.0-30.6)	21.3 (20.0-22.6)	28.5 (27.2-29.7)	23.7 (22.3-25.2)
Girls	18.6 (11.8-28.0)	16.5 (15.4-17.8)	23.1 (21.5-24.7)	19.3 (17.5-21.2)
Boys	31.9 (26.8-37.6)^G,W^	25.7 (24.0-27.6)	33.8 (32.0-35.6)	28.0 (26.2-29.8)
**Did not attend physical education classes^g^**				
Total	46.7 (40.2-53.3)^H^	47.7 (44.2-51.3)	43.7 (40.4-47.0)	39.9 (37.0-42.8)
Girls	49.3 (39.2-59.4)	50.7 (46.9-54.6)	49.3 (45.3-53.3)	43.1 (39.8-46.5)
Boys	44.3 (37.8-51.0)^H^	44.8 (41.1-48.6)	37.9 (34.9-40.9)	36.6 (33.7-39.6)
**Did not play on ≥1 sports team^h^**				
Total	49.0 (44.4-53.7)^W^	40.8 (39.3-42.3)	45.5 (44.0-47.0)	48.9 (47.6-50.3)
Girls	53.3 (46.8-59.7)^W^	44.9 (43.3-46.5)	56.6 (54.7-58.5)	57.2 (55.5-58.8)
Boys	45.1 (39.1-51.2)^G,W,B^	36.8 (34.9-38.8)	34.0 (32.4-35.8)	40.6 (39.1-42.3)

Abbreviations: AI/AN, American Indian/Alaska Native; CI, confidence interval.

a Non-Hispanic.

b The letter W (white), B (black), or H (Hispanic) indicates a significant difference in prevalence estimates between AI/AN students and students of that race/ethnicity. The letter G indicates a significant difference in prevalence estimates between AI/AN girls and boys (*P* ≤ .05, *t* test).

c Exercised or participated in physical activities that made students sweat and breathe hard for at least 20 minutes on less than 3 of the 7 days before the survey (eg, basketball, soccer, running, swimming laps, fast bicycling, fast dancing, similar aerobic activities).

d Physical activities that did not make students sweat and breathe hard for at least 30 minutes on less than 5 of the 7 days before the survey (eg, fast walking, slow bicycling, skating, pushing a lawn mower, mopping floors).

e On an average school day.

f Played video or computer games or used a computer for something that was not school work.

g On 1 or more days in an average week when they were in school.

h Run by their school or community groups during the 12 months before the survey.

**Table 4:** Selected *Healthy People 2010* Objectives and Targets for Adolescents

**Objective Number**	Objective	Target
19-3b	Reduce the proportion of children and adolescents who are overweight or obese (adolescents aged 12-19 y)	5%
19-5	Increase the proportion of persons aged 2 years and older who consume at least 2 daily servings of fruit	75%
19-6	Increase the proportion of persons aged 2 years and older who consume at least 3 daily servings of vegetables, with at least one-third being dark green or orange vegetables	50%
19-11	Increase the proportion of persons aged 2 years and older who meet dietary recommendations for calcium	75%
22-6	Increase the proportion of adolescents who engage in moderate physical activity for at least 30 minutes on 5 or more of the previous 7 days	35%
22-7	Increase the proportion of adolescents who engage in vigorous physical activity that promotes cardiorespiratory fitness 3 or more days per week for 20 or more minutes per occasion	85%
22-9	Increase the proportion of adolescents who participate in daily school physical education	50%
22-11	Increase the proportion of adolescents who view television 2 or fewer hours on a school day	75%
26-11d	Reduce the proportion of persons engaging in binge drinking of alcoholic beverages (adolescents aged 12-17 y)	2%
27-2b	Reduce tobacco use by adolescents (cigarettes in the past month by students in grades 9 through 12)	16%
